# Intrazerebrale Blutungen unter Plättchenaggregationshemmung und oraler Antikoagulation bei Patienten mit zerebraler Amyloidangiopathie

**DOI:** 10.1007/s00115-021-01206-w

**Published:** 2021-10-15

**Authors:** R. Haußmann, P. Homeyer, M. Haußmann, M. Brandt, M. Donix, V. Puetz, J. Linn

**Affiliations:** 1 Klinik und Poliklinik für Psychiatrie und Psychotherapie, Universitätsklinikum Carl Gustav Carus, Technische Universität Dresden, Fetscherstr. 74, 01307 Dresden, Deutschland; 2 Dialysepraxis Leipzig, MVZ, Plantagenweg 2, 04178 Leipzig, Deutschland; 3 Klinik und Poliklinik für Neurologie, Universitätsklinikum Carl Gustav Carus, Technische Universität Dresden, Fetscherstr. 74, 01307 Dresden, Deutschland; 4 Institut und Poliklinik für diagnostische und interventionelle Neuroradiologie, Universitätsklinikum Carl Gustav Carus, Technische Universität Dresden, Fetscherstr. 74, 01307 Dresden, Deutschland; 5 DZNE, Deutsches Zentrum für Neurodegenerative Erkrankungen, Dresden, Deutschland; 6 DNVC, Dresdner Neurovaskuläres Centrum, Dresden, Deutschland; 7 Universitäts DemenzCentrum (UDC), Klinik und Poliklinik für Psychiatrie und Psychotherapie, Uniklinikum Dresden, Dresden, Deutschland

**Keywords:** Cerebrale Amyloidangiopathie, Intracerebrale Blutung, Thrombozytenaggregationshemmung, Orale Antikoagulation, Interventioneller Vorhofohrverschluss, Cerebral amyloid angiopathy, Intracerebral hemorrhage, Platelet inhibition, Oral anticoagulation, Interventional left atrial appendage closure

## Abstract

Die Durchführung einer oralen Antikoagulation ist bei Patienten mit einer zerebralen Amyloidangiopathie eine therapeutische Herausforderung. Die Assoziation der zerebralen Amyloidangiopathie mit Lobärblutungen, eine hohe Mortalität intrazerebraler Blutungen insbesondere unter oraler Antikoagulation sowie das hohe Rezidivrisiko solcher Blutungen erfordern eine strenge und interdisziplinäre Risiko-Nutzen-Abwägung. Vitamin-K-Antagonisten erhöhen das Risiko für die mit intrazerebralen Blutungen vergesellschaftete Mortalität um 60 % und sollten daher möglichst vermieden bzw. speziellen klinischen Situationen (z. B. mechanischer Aortenklappenersatz) vorbehalten sein. Auch der Einsatz von neuen oralen Antikoagulanzien und Thrombozytenaggregationshemmern bedarf einer strengen Risiko-Nutzen-Abwägung, da auch diese Substanzen das zerebrale Blutungsrisiko erhöhen. Insbesondere bei Patienten mit einer absoluten Arrhyhtmie bei Vorhofflimmern ist der interventionelle Vorhofohrverschluss eine therapeutische Alternative. Darüber hinaus sind weitere klinische Implikationen bei Patienten mit zerebraler Amyloidangiopathie Gegenstand dieser Literaturübersicht, beispielsweise Besonderheiten nach akutem ischämischem Schlaganfall und erforderlicher Sekundärprophylaxe, bei vorherigen intrazerebralen Blutungen und bei Patienten mit kognitiven Defiziten.

## Abstract (en)

Oral anticoagulation in patients with cerebral amyloid angiopathy is a therapeutic challenge. The association of cerebral amyloid angiopathy with intracerebral hemorrhage, a high mortality of intracerebral hemorrhage especially under oral anticoagulation and the high risk of recurrent bleeding require a multidisciplinary approach and a thorough risk-benefit analysis. Vitamin K antagonists increase the risk of intracerebral bleeding and the accompanying mortality by 60% and should be avoided if possible or reserved for special clinical situations (e.g. mechanical aortic valve replacement). Treatment with novel oral anticoagulants and antiplatelet drugs also increases the risk of cerebral bleeding and therefore needs a thorough risk-benefit evaluation. An interventional left atrial appendage closure is a promising therapeutic option especially in patients with an absolute arrythmia with atrial fibrillation. Furthermore, other clinical implications in patients with cerebral amyloid angiopathy are the subject of this review of the literature, such as special characteristics after acute ischemic stroke and the necessary secondary prophylaxis, with previous intracerebral hemorrhage and in patients with cognitive deficits.

## Hintergrund

Eine zerebrale Amyloidangiopathie (CAA) ist bei 20–40 % der älteren Bevölkerung ohne Demenzerkrankung und bei 50–60 % der Menschen mit Demenzerkrankung nachweisbar [1]. Die CAA kann sich mit intrazerebralen Lobärhämatomen, zerebralen Mikroblutungen, fokalen Subarachnoidalblutungen und einer kortikalen superfiziellen Siderose (cSS) manifestieren. Bei der cSS handelt es sich um lineare Hämosiderinablagerungen im Subarachnoidalraum oder den oberflächlichen Kortexschichten der Konvexität, die Residuen vorangegangener fokaler Subarachnoidalblutungen darstellen. Die CAA wird bei einem beträchtlichen Anteil der Fälle nichttraumatischer intrazerebraler Blutungen (ICB) als ursächlich angenommen [1]. Patienten mit einer CAA und stattgehabter ICB haben ferner ein erhöhtes Rezidivrisiko für eine erneute ICB [2]. Die bedeutsamsten Prädiktoren für die Entwicklung einer ICB bei Patienten mit einer CAA sind eine zurückliegende ICB, das Vorliegen einer disseminierten cSS sowie die Dauer und Art der oralen Antikoagulation [2]. Insbesondere bei bestehender Indikation zur therapeutischen Antikoagulation älterer Patienten, beispielsweise aufgrund eines Vorhofflimmerns, sehen sich Behandler häufig mit dem Dilemma konfrontiert, dass die aktuell verfügbaren Assessments zur Abschätzung des Blutungsrisikos unter Antikoagulation, wie beispielsweise der HAS-BLED-Score [3], das Vorliegen einer CAA und das damit assoziierte ICB-Risiko unzureichend berücksichtigen und momentan eine geringe Evidenz zur Risiko-Nutzen-Abwägung hinsichtlich der Einleitung oder des Wiederbeginns einer oralen Antikoagulation bei diesen Patienten besteht.

Etwa 9 % der über 65-jährigen Menschen leiden an einer absoluten Arrhythmie bei Vorhofflimmern, und sowohl die Prävalenz der CAA als auch die Prävalenz des Vorhofflimmerns steigen im höheren Lebensalter deutlich an [4, 5], weshalb dieses Dilemma von wachsender klinischer Bedeutung ist. Vor diesem Hintergrund fasst diese Literaturübersichtsarbeit aktuelle Daten zum Blutungsrisiko bei Patienten mit CAA und gerinnungshemmender Medikation zusammen.

## Methodik

Diese Übersichtsarbeit wurde unter Nutzung der über PubMed verbundenen biomedizinischen Datenbanken erstellt. Eine zusätzliche Suche erfolgte anhand der in den Literaturverzeichnissen gefundenen Original- und Übersichtsarbeiten. Dabei wurden präferenziell Originalarbeiten gesucht. Auch die verfügbaren Leitlinien wurden berücksichtigt. In Anbetracht der Datenlage fanden jedoch verschiedene Studiendesigns, metaanalytische Daten und auch Literaturübersichten Berücksichtigung.

## Risiko für intrazerebrale Blutungen bei zerebraler Amyloidangiopathie allgemein

Die CAA wird zunehmend als wichtige Ursache für eine lobäre ICB im höheren Lebensalter wahrgenommen [6]. Das primäre ICB-Risiko bei Patienten mit einer CAA ist abhängig von verschiedenen MRT-Biomarkern und von der allgemeinen Amyloidlast [4]. Lobäre Hämorrhagien auf dem Boden einer CAA weisen eine Mortalität von 20–30 % auf [7, 8]. Insbesondere ICBs unter oraler Antikoagulation sind mit einer hohen Mortalität von bis zu 52 % vergesellschaftet [4]. Eine Hämatomgröße < 50 ml und eine GCS-Score ≥ 8 sind in diesem Zusammenhang bedeutsame Prädiktoren für eine günstige Prognose [7].

Patienten mit einer CAA und vorheriger ICB weisen ein jährliches Rezidivblutungsrisiko von 8,9 % pro Jahr auf [4]. Insbesondere im Vergleich zu Patienten mit einer hypertensiv bedingten ICB weisen Patienten mit einer CAA-assoziierten ICB ein vergleichsweise hohes Rezidivrisiko auf [9]. In zwei Fallserien lag die Rezidivblutungsrate nach 2 Jahren bei 21 % bzw. nach 2,6 Jahren bei 24 % [10, 11]. Andere Studien zeigen ein ungefähres Rezidivblutungsrisiko bei hypertensiver ICB von 7 % [12]. Aktuelle Daten lassen vermuten, dass das Rezidivblutungsrisiko wiederum mit jeder weiteren ICB ansteigt [13]. Insbesondere Areale mit hoher Amyloidlast scheinen dabei Prädilektionsstellen für weitere ICBs zu sein [8, 14].

Als Risikofaktoren für eine ICB im Rahmen einer CAA gelten eine stattgehabte ICB (HR 7,7; 95 %-KI 1,4–15,7), das Vorliegen einer cSS, eine höhere Anzahl lobärer Mikroblutungen und CT-Hypodensitäten im posterioren Marklager [13]. Das mit kortikalen Mikroblutungen assoziierte ICB-Risiko nimmt mit zunehmender Anzahl der Mikroblutungen zu [4]. Bezüglich der cSS konnte eine Metaanalyse von 6 Studien mit > 1200 symptomatischen Patienten zeigen, dass das jährliche ICB-Risiko bei Patienten in Abhängigkeit des Vorliegens und Schweregrades einer cSS ansteigt: ohne cSS 3,9 % pro Jahr (95 %-KI 1,7–6,1 %), mit cSS 11,1 % pro Jahr (95 %-KI 7–15,2 %; [15]). Bei Patienten mit einer fokalen cSS (< 4 betroffene kortikale Furchen) betrug das ICB-Risiko 9,1 % pro Jahr, während es bei den Patienten mit einer disseminierter cSS (≥ 4 Furchen) 12,5 % pro Jahr betrug [15]. Somit ist neben dem Vorliegen einer cSS auch der Ausprägungsgrad (fokal vs. disseminiert) als zusätzlicher Risikofaktor für eine ICB zu betrachten (HR fokale cSS: 2,11 [KI] vs. HR disseminierte cSS: 4,28 [KI]; [15]). Andere Daten zeigen ein jährliches Blutungsrisiko von 19 % pro Jahr bei Patienten mit CAA und zurückliegender ICB, wenn bildmorphologisch fokale Subarachnoidalblutungen (fSAH), d. h. umschriebene Subarachnoidalblutungen im Bereich der Konvexitäten der Großhirnhemisphären, gefunden werden [4].

## Risiko für intrazerebrale Blutungen bei CAA unter Thrombozytenaggregationshemmung

Die Therapie mit Thrombozytenaggregationshemmern war in einer populationsbasierten Querschnittstudie mit 1062 Patienten > 60 Jahre mit einer höheren Prävalenz zerebraler Mikroblutungen assoziiert [16]. Patienten unter antithrombotischer Medikation wiesen mehr zerebrale Mikroblutungen auf als Patienten ohne thrombozytenaggregationshemmende Therapie (adjustierte OR 1,71; 95 %-KI 1,21–2,41), wobei dieser Zusammenhang für orale Antikoagulanzien in der untersuchten Kohorte nicht gefunden wurde (adjustierte OR 1,49; 95 %-KI 0,82–2,71; [16]). Insbesondere die Häufigkeit strikt lobär lokalisierter zerebraler Mikroblutungen war unter Behandlung mit ASS erhöht, was eine differenzielle Beeinflussung des Blutungsrisikos bei Patienten mit einer CAA suggeriert [16]. Um das „Confounding-by-indication“-Bias möglichst gering zu halten, erfolgten in dieser Analyse eine Adjustierung für das kardiovaskuläre Risiko und ein Ausschluss von Patienten mit bekannter zerebrovaskulärer Erkrankung.

Nach stattgehabter ICB und nach Kontrolle für sonstige klinische ICB-Prädiktoren erhöht ASS in multivariaten Analysen das Risiko für eine Rezidivblutung (HR 3,95, 95 %-KI 1,6–8,3; *p* = 0,021; [13]). Bei Patienten nach ICB war der Wiederbeginn einer Thrombozytenaggregationshemmung bei Patienten mit einer CAA jedoch nicht mit einem erhöhten Rezidiv-ICB-Risiko vergesellschaftet [17]. Relevante Limitationen der bisherigen Daten zu Assoziationen zwischen zerebralen Mikroblutungen und bestehender Thrombozytenaggregationshemmung sind das oft retrospektive Studiendesign, welches die Beschreibung von Kausalzusammenhängen begrenzt, die Tatsache, dass Hämosiderinablagerungen für eine nicht näher definierte Zeitdauer nachweisbar bleiben und somit möglicherweise vorbestehende Mikroblutungen in den durchgeführten Untersuchungen Ergebnisse zu entsprechenden Assoziationen verfälschen sowie eine Verzerrung dadurch, dass insbesondere Patienten mit erhöhtem kardiovaskulärem Risiko, die a priori auch ein erhöhtes Risiko für zerebrale Mikroblutungen aufweisen, eine thrombozytenenaggregationshemmende Therapie erhalten [16]. Eine kanadische Machbarkeitsstudie in 2 Phasen (NASPAF-ICH; 1. Phase: Ausschluss von Patienten mit CAA, 2. Phase Einschluss von Patienten mit CAA) mit den kombinierten Endpunkten ischämischer Schlaganfall und ICB nach Wiederbeginn von ASS oder NOAK zeigte bei bislang 30 eingeschlossenen Patienten (9 mit ASS, 21 mit NOAK) unter striktem Blutdruckprotokoll (RR < 130/80) keine erneuten ICBs bei einem ischämischen Schlaganfall in der ASS-Gruppe [18, 19].

## Risiko für intrazerebrale Blutungen unter oraler Antikoagulation

In einer Untersuchung von 6045 Patienten > 55 Jahre mit Vorhofflimmern und durchschnittlich 6‑jähriger Behandlungsdauer mit oraler Antikoagulation oder Thrombozytenaggregationshemmung entwickelten 74 Patienten (1,22 %) eine nichttraumatische ICB, von denen 51,4 % die diagnostischen Kriterien einer CAA erfüllten [20]. Die Mortalität zwischen Patienten mit und ohne Zeichen einer CAA unterschied sich nicht (orale Antikoagulanzien: 10-Jahres-Mortalität CAA-Gruppe 45 % vs. 63 % in Nicht-CAA-Gruppe [*p* = 0,46]; Thrombozytenaggregationshemmung: 10-Jahres-Mortalität CAA-Gruppe 79 % vs. 70 % Nicht-CAA-Gruppe [*p* = 0,66]). Vor allem unter Therapie mit Vitamin-K-Antagonisten ist das Auftreten einer ICB mit dem Vorhandensein zerebraler Mikroblutungen assoziiert, was eine Risikoerhöhung für Vitamin K‑Antagonisten-assoziierte ICBs suggeriert [21]. Das höchste Risiko besteht unter Therapie mit Phenprocoumon bzw. Warfarin, gefolgt von neuen oralen Antikoagulanzien (NOAK) und Thrombozytenaggregationshemmern [2]. Insbesondere die orale Antikoagulation mit Vitamin-K-Antagonisten erhöht das ICB-Risiko bei Patienten mit einer CAA um den Faktor 7 bis 10 und die ICB-assoziierte Mortalität um 60 % [22–24]. Eine prospektive Beobachtungsstudie bei Patienten mit neu diagnostiziertem Vorhofflimmern, neu begonnener oraler Antikoagulation, Baseline-Schädel-MRT und einem Follow-up nach 24 Monaten mit 1490 Patienten (CROMIS-2-Studie) zeigte insgesamt 14 ICBs im Beobachtungszeitraum (11 hemisphärielle Blutungen, 1 Subduralhämatom, 1 Subarachnoidalblutung; [25]). Im initialen MRT wiesen 21 % kortikale Mikroblutungen und < 1 % (*n* = 5) eine cSS auf; 3 % erfüllten die modifizierten Boston-Kriterien für die Diagnose einer wahrscheinlichen CAA. Die Patienten der ICB-Gruppe litten häufiger an einem Diabetes mellitus, waren häufiger auf einen Vitamin-K-Antagonisten als auf NOAK eingestellt (12 vs. 2) und wiesen mehr kortikale Mikroblutungen und cSS auf. Insbesondere das Vorliegen von Mikroblutungen erhöhte das Blutungsrisiko im Vergleich zu Patienten ohne kortikale Mikroblutungen um den Faktor 3. Diese Untersuchung liefert einen wichtigen Beitrag zur besseren Prädiktion einer ICB im Rahmen eines kombinierten Modells unter Berücksichtigung von HAS-BLED-Scores, kortikalen Mikroblutungen, Diabetes mellitus und Art der Antikoagulation.

## Klinische Implikationen

Vor dem Hintergrund des erhöhten Risikos für spontane Lobärblutungen bei Patienten mit einer CAA unter oraler Antikoagulation bedarf es einer individualisierten Risiko-Nutzen-Abwägung vor der Behandlung mit Thrombozytenaggregationshemmern und oralen Antikoagulanzien. Entscheidend im Rahmen dieser Abwägung sind stets die Stärke der jeweiligen Indikation, das individuelle ICB-Risiko sowie die Verfügbarkeit alternativer Therapieoptionen [2]. In derartigen Situationen sollten daher bestehende Risikofaktoren für intrazerebrale Blutungen und thrombembolische Ereignisse gegenübergestellt und eine individuelle Nutzen-Risiko-Abwägung getroffen werden (Tab. 1).

**Table 1:** 

Risikofaktoren für ICB bei CAA	Risikofaktoren für thrombembolische Komplikationen
Z. n. ICB	Tumorassoziierte und andere Hyperkoagulopathien
Disseminierte kortikale superfizielle Siderose	Z. n. mechanischem Klappenersatz
Antikoagulation mit Phenprocoumon, (N)OAK, NSAR – insbesondere bei längerer Therapiedauer	(Z. n.) tiefe(r) Beinvenenthrombose
	Vorhof- oder Ventrikelthrombus
Hohe Anzahl lobärer Mikroblutungen	Vorhofflimmern
Hohe Amyloidlast	Z. n. zerebraler Ischämie

Insbesondere beim komorbiden Vorliegen einer CAA und einer absoluten Arrhythmie bei Vorhofflimmern ist das therapeutische Management Gegenstand kontroverser Diskussionen und bislang mit unzureichender Evidenz unterfüttert [26]. Der interventionelle Verschluss des linken Vorhofohres stellt eine mögliche therapeutische Alternative dar. Kleinere Beobachtungsstudien und eine Metaanalyse von 19 RCTs aus dem Jahre 2017 deuten darauf hin, dass es sich beim Vorhofohrverschluss um eine therapeutische Maßnahme mit einer ähnlichen Effektivität wie die der oralen Antikoagulation handelt [26, 27]. Die 5‑Jahres-Nachbeoachtung der PROTECT-AF- und PREVAIL-Studie, die die Wirksamkeit von Vitamin-K-Antagonisten und interventionellem Vorhofohrverschluss verglichen, zeigte signifikant weniger ICBs bei einer vergleichbaren Prävalenz ischämischer Schlaganfälle [28].

## Wiederbeginn einer oralen Antikoagulation nach ICB

Bei Patienten nach stattgehabter ICB unter Antikoagulanzientherapie stellt sich im klinischen Kontext häufig die Frage, ob eine vorbestehende orale Antikoagulation wieder aufgenommen werden sollte. Neben einem unzureichend behandelten Hypertonus, einem hohen Lebensalter, der Notwendigkeit einer dualen Thrombozytenaggregationshemmung und einer erlittenen ICB sprechen auch multiple zerebrale Mikroblutungen gegen einen Wiederbeginn der oralen Antikoagulation [1]. Sowohl für Vitamin-K-Antagonisten als auch für NOAKs stellt eine zurückliegende ICB formal eine Kontraindikation für eine weitere Gabe dar. Dennoch profitieren Patienten auch in dieser klinischen Konstellation wahrscheinlich vom Wiederbeginn der oralen Antikoagulation und auch vom Wiederbeginn einer vorbestehenden Thrombozytenaggregationshemmung [17, 29].

Eine Metaanalyse mit Daten aus drei Registerstudien mit insgesamt 633 Patienten mit nichtlobärer und 379 Patienten mit lobärer antikoagulanzienassoziierter ICB zeigte, dass die Wiederaufnahme der oralen Antikoagulation mit einer signifikant geringeren Mortalität, einem besseren klinisch-funktionellen Outcome und einer signifikant reduzierten Rate ischämischer Schlaganfälle bei nicht erhöhtem ICB-Risiko, sowohl hinsichtlich kortikaler als auch hinsichtlich subkortikaler ICBs, assoziiert ist [30].

Der Nachweis einer CAA kann alternativ dazu führen, dass von einer erneuten oralen Antikoagulation abgesehen und ein Vorhofohrverschluss präferiert wird [1]. In welchem Ausmaß Patienten mit Vorhofflimmern nach einer stattgehabten ICB von einem interventionellen Vorhofohrverschluss profitieren, ist zum aktuellen Zeitpunkt jedoch nicht geklärt und Gegenstand laufender prospektiver Studien (z. B. STROKECLOSE-Studie; [31]).

Darüber hinaus sind begleitende kognitive Defizite bei bestehender Indikation zur oralen Antikoagulation zu berücksichtigen. Etwa 25 % älterer Patienten mit Vorhofflimmern weisen bereits vor der ersten ICB kognitive Defizite auf [32], welche wiederum auch Assoziationen zur CAA aufweisen. Insbesondere bei Patienten mit Alzheimer-Demenz weisen Mikroblutungen auf eine Assoziationen zum Vorliegen einer CAA hin [33]. Aus diesem Grund sollten Patienten mit kognitivem Abbau, vorheriger ICB oder transienten Episoden fokal-neurologischer Defizite vor Beginn der Antikoagulation eine MRT des Neurokraniums erhalten, um das Vorhandensein kortikaler Mikroblutungen, einer cSS oder einer sSAH als mögliche Korrelate einer CAA zu erkennen [4]. Im Rahmen eines multidisziplinären Assessments und unter Berücksichtigung der Erkenntnisse aus der CROMIS-2-Studie sollte eine ausführliche Risiko-Nutzen-Abwägung hinsichtlich der Eindosierung einer oralen Antikoagulation erfolgen. Beim Vorliegen einer CAA sollten Vitamin-K-Antagonisten zugunsten des Einsatzes von NOAKs vermieden werden, sofern keine spezielle Indikation für den Einsatz von Vitamin-K-Antagonisten besteht (z. B. bei valvulärem Vorhofflimmern, Z. n. mechanischem Herzklappenersatz). Wenn der Einsatz eines NOAK klar indiziert ist, bedarf es vor allem bei älteren Patienten einer Reevaluation des Blutungsrisikos, insbesondere in Form eines engmaschigen Blutdruckmonitorings mit einem Blutdruckziel unter 130/80 mm Hg und gegebenenfalls einer zerebralen MRT-Kontrolle im Verlauf [4].

## Weitere therapeutische Aspekte

Das Vorliegen einer CAA muss außerdem bei der akuten Schlaganfallbehandlung und der Sekundärprävention gewürdigt werden [34]. Das Vorhandensein und eine hohe Last an zerebralen Mikroblutungen sind unabhängige Prädiktoren für eine symptomatische ICB unter i.v. Thrombolysetherapie [35]. Insbesondere eine hohe Last an zerebralen Mikroblutungen ist im Kontext der i.v. Thrombolysetherapie mit einer erhöhten Mortalität und insbesondere bei älteren Patienten mit dem Auftreten lyseassoziierter ICBs verbunden [36], wobei das Vorhandensein von Mikroblutungen nach jetziger Studienlage trotz des erhöhten ICB-Risikos keine Kontraindikation für eine systemische Thrombolyse darstellt. In Grenzsituationen (z. B. geringes klinisches Defizit) kann das bekannte Vorliegen einer CAA jedoch nach unserer Ansicht in die individualisierte Entscheidungsfindung und Nutzen-Risiko-Abwägung zur Durchführung einer systemischen Lysetherapie einfließen.

Unter i.v. Thrombolysetherapie neu aufgetretene zerebrale Mikroblutungen sind annehmbar mit dem Vorliegen einer CAA vergesellschaftet [37]. Aktuell existieren jedoch keine gesonderten leitlinienbasierten Empfehlungen zur Behandlung des ischämischen Schlaganfalls bei Patienten mit schwerer Mikroangiopathie, Amyloidangiopathie oder Vorbehandlung mit Thrombozytenaggregationshemmern.

Im Bereich der Sekundärprävention ischämischer Schlaganfälle zeigen aktuelle Daten, dass eine konsequente antihypertensive Therapie mit einem Zielblutdruck < 130/80 mm Hg das Risiko für eine Rezidiv-ICB bei Patienten mit einer CAA deutlich reduzieren kann [38]. Ferner gilt es, aktuelle Daten hinsichtlich einer sekundärprophylaktischen Therapie mit Statinen zu berücksichtigen. Während ein allgemeiner Konsens hinsichtlich einer Inzidenzreduktion kardiovaskulärer Ereignisse unter Statintherapie besteht, wurde eine erhöhte Inzidenz von ICBs und insbesondere kortikosubkortikaler Mikroblutungen beobachtet, was auf eine mögliche Assoziationen zur CAA hindeutet [34, 39]. Es wird kontrovers diskutiert, ob das Risiko einer Rezidivblutung nach ICB unter einer sekundärprophylaktischen Statintherapie ansteigt [40]. Neuere Daten zeigen, dass Statine bei Patienten nach Schlaganfall das ICB-Risiko zwar erhöhen, die präventiven Effekte hinsichtlich ischämischer Schlaganfälle insgesamt jedoch zu überwiegen scheinen [41]. Möglicherweise führt die frühe Initiierung einer Statintherapie bei Patienten mit lobärer ICB zu einer Vergrößerung des perihämorrhagischen Ödems [42]. Es bedarf jedoch weiterer Studien, um Statineffekte auf das ICB-Risiko differenziert beschreiben und antizipieren zu können.

## Fazit für die Praxis

Das erhöhte ICB-Rezidivrisiko bei Patienten mit einer CAA unter Behandlung mit Thrombozytenaggregationshemmern und oralen Antikoagulanzien muss im klinischen Kontext Berücksichtigung finden. Klinische Szenarien, in denen eine bestehende Indikation zur oralen Antikoagulation mit dem Blutungsrisiko bei komorbid bestehender CAA abgewogen werden muss, bedürfen eines multidisziplinären Assessments und einer interdisziplinären Risiko-Nutzen-Abwägung unter Würdigung der individuellen Risikofaktoren für ICBs und thrombembolische Ereignisse. Da die Datenlage aktuell begrenzt ist, bleibt die Entscheidung für oder gegen eine orale Antikoagulation bei Patienten mit CAA zum gegenwärtigen Zeitpunkt eine Einzelfallentscheidung. Der interventionelle Vorhofohrverschluss kann in derartigen Situationen eine therapeutische Alternative darstellen. Außerdem sollte das Blutungsrisiko im Verlauf der Behandlung älterer Patienten kontinuierlich reevaluiert werden. Bei Patienten nach ICB, mit kognitivem Abbau oder zurückliegenden transienten Episoden neurologischer Defizite sollte vor Beginn der oralen Antikoagulation eine MRT des Neurokraniums erfolgen.
